# Ultrasonographic optic nerve sheath diameter as a non-invasive marker of intracranial hypertension in pediatric patients: a pilot study

**DOI:** 10.3389/fped.2026.1785962

**Published:** 2026-04-29

**Authors:** Raúl Montero-Yéboles, Alberto Ferrin-Diánez, Patricia Roselló, Jose Luis López-Prats, Beatriz Ruiz-Sáez, José Luis Vázquez

**Affiliations:** 1 1Pediatric Intensive Care Unit, Reina Sofía Universitary Hospital, Córdoba, Spain; 2 Pediatric Intensive Care Unit, Clinico Universitary Hospital, Valencia, Spain; 3 Pediatric Intensive Care Unit, Ramon y Cajal Universitary Hospital, Madrid, Spain

**Keywords:** intracranial hypertension, intracranial pressure, non invasive monitoring, optic nerve sheath diameter, pediatrics, point of care ultrasound, ultrasound

## Abstract

**Background:**

Intracranial hypertension (IH) is a potentially life-threatening condition in pediatric patients that requires early recognition. Ultrasonographic measurement of optic nerve sheath diameter (ONSD) has emerged as a promising non-invasive surrogate for intracranial pressure (ICP), although pediatric data remain limited and diagnostic thresholds are not well established.

**Objective:**

To evaluate the correlation between ONSD and directly measured ICP, and to determine an optimal ONSD cutoff for detecting intracranial hypertension in pediatric patients with closed fontanelles.

**Methods:**

This prospective observational pilot study included pediatric patients admitted to a tertiary care center who required lumbar puncture with ICP measurement. ONSD was measured using transocular ultrasound immediately prior to lumbar puncture, at 3 mm posterior to the globe. Mean bilateral ONSD values were used when available. Correlation between ONSD and ICP was assessed using Spearman's rho. Receiver operating characteristic (ROC) curve analysis was performed to determine the optimal cutoff for detecting intracranial hypertension (defined as ICP >27 cmH₂O).

**Results:**

Thirteen patients were included, yielding 15 paired ONSD–ICP measurements. Median ONSD was 6.20 mm (IQR 5.68–6.53), and median ICP was 34 cmH₂O (IQR 26–44). A significant positive correlation was observed between ONSD and ICP (Spearman's rho = 0.65, *p* = 0.009). ROC analysis identified an optimal ONSD cutoff of 6.1 mm, with a sensitivity of 80% and specificity of 80% (AUC 0.88). Agreement between ONSD and ICP classification was moderate (*κ* = 0.57).

**Conclusion:**

ONSD ultrasonography appears to be a feasible and promising non-invasive method for detecting intracranial hypertension in pediatric patients with closed fontanelles. An ONSD threshold of 6.1 mm may help identify elevated ICP, although larger multicenter studies are required to validate these findings

## Introduction

Intracranial hypertension (IH) is a potentially life-threatening condition in paediatric patients and requires early recognition and prompt management. According to the Monro–Kellie doctrine, the cranial vault contains brain parenchyma, cerebrospinal fluid (CSF), and blood, and an imbalance among these components results in increased intracranial pressure (ICP), reduced cerebral perfusion pressure, and impaired cerebral blood flow, leading to secondary brain injury (1, 2).

Direct measurement of ICP remains the diagnostic gold standard; however, it is invasive, associated with potential complications, and not always feasible in children (3). Neuroimaging techniques may support the diagnosis of IH but often require patient transport and, in younger children, sedation, which can be challenging in the pediatric intensive care setting (4). Consequently, there is growing interest in non-invasive bedside techniques that allow early detection of IH in critically ill pediatric patients.

The optic nerve is surrounded by a dural sheath that is anatomically continuous with the intracranial subarachnoid space. Increases in CSF pressure are transmitted along this space, resulting in distension of the optic nerve sheath (5). Ultrasonographic measurement of the optic nerve sheath diameter (ONSD) has therefore emerged as a promising non-invasive surrogate marker of elevated ICP. Experimental and anatomical studies have demonstrated that optic nerve sheath distension is most pronounced in the anterior retrobulbar segment, approximately 3 mm posterior to the globe, which has become the standard measurement site (5, 6).

In adult populations, several clinical studies have demonstrated good diagnostic accuracy of ONSD ultrasound for detecting elevated ICP in both traumatic and non-traumatic brain injury (7, 8). A large meta-analysis further supported the utility of ONSD measurement as a screening tool for intracranial hypertension, reporting high sensitivity and specificity across diverse clinical settings (9).

In contrast, paediatric data remain limited, and reported diagnostic thresholds for ONSD vary widely depending on age, ultrasound technique, and reference standard used for ICP assessment. Previous paediatric studies have proposed cutoff values ranging from 4.0 to 5.5 mm, with higher thresholds generally reported in older children with closed fontanelles (10–12). As a result, the optimal ONSD threshold for detecting intracranial hypertension in paediatric patients has not yet been clearly established.

The aim of this prospective pilot study was to evaluate the correlation between ultrasonographic ONSD measurements and simultaneously obtained direct ICP measurements in a paediatric population and to determine an ONSD cutoff value for the detection of intracranial hypertension in children with closed fontanelles.

## Materials and methods

### Study design and population

This prospective observational pilot study was conducted at a single tertiary paediatric center between January 2022 and April 2025. Paediatric patients admitted to the paediatric intensive care unit who required lumbar puncture with direct ICP measurement were eligible. Due to the low incidence of paediatric intracranial hypertension and the relatively small paediatric catchment area of our institution, only a limited number of eligible cases were expected during the study period. Therefore, all consecutive patients meeting inclusion criteria were enrolled, and no formal sample size calculation was performed because the study was designed as an exploratory prospective cohort.

### Inclusion and exclusion criteria

Inclusion criteria were age <18 years and indication for lumbar puncture with ICP measurement. Exclusion criteria included age ≥18 years, presence of exophthalmos or thyroid eye disease, and known intracranial hypertension within the previous six months due to space-occupying lesions.

### Optic nerve sheath ultrasound

Optic nerve sheath diameter (ONSD) measurements were performed immediately prior to lumbar puncture using a Sonoscape ultrasound system with a 10-MHz linear probe following a standardized transocular ultrasound protocol. To minimize temporal variability between measurements, ultrasound assessment was performed at the bedside immediately before cerebrospinal fluid opening pressure measurement.

Patients were positioned in the lateral decubitus position for both procedures. ONSD measurements were obtained with the patient in the same position used for lumbar puncture to maintain consistency between assessments.

Measurements were obtained 3 mm posterior to the vitreoretinal junction, perpendicular to the optic nerve axis. Two to three measurements were obtained in each eye and mean values were calculated.

In a small number of examinations, bilateral measurements were not feasible due to practical limitations during bedside image acquisition, including patient agitation, limited acoustic window, or time constraints related to the clinical procedure. In these cases, unilateral measurements were used for analysis.

### Intracranial pressure measurement

Direct ICP measurement was performed via lumbar puncture using a sterile disposable manometer (Becton Dickinson Manometer Kit REF 4330). Lumbar puncture was performed with patients in the lateral decubitus position to ensure standardized pressure measurement. ICP values were recorded in cmH₂O.

Intracranial hypertension was defined as ICP >27 cmH₂O (≈20 mmHg). Although some variability exists regarding the optimal diagnostic threshold, this value has been widely adopted in clinical practice and consensus recommendations as a practical cutoff for defining intracranial hypertension. Slightly higher thresholds have been proposed for therapeutic decision-making in severe brain injury, but given the limited evidence supporting those values, we selected 20 mmHg as the operational definition for this study.

### Statistical analysis

Statistical analysis was performed using SPSS software. Inter-eye differences were assessed using the Wilcoxon test. Correlation between ONSD and ICP was analyzed using Spearman's rho. Receiver operating characteristic (ROC) curve analysis was used to identify the optimal ONSD cutoff value for detecting intracranial hypertension. Agreement between methods was assessed using Cohen's kappa. This study was conducted and reported in accordance with STROBE guidelines for observational studies..

## Results

Thirteen pediatric patients were included in the study, yielding a total of 15 paired ONSD–ICP measurements. Two patients underwent repeated lumbar punctures during their clinical course, resulting in additional paired observations. The median age of the cohort was 11.8 years (IQR 8–13.5), and seven patients were male. All patients had closed fontanelles.

Individual paired optic nerve sheath diameter (ONSD) and intracranial pressure (ICP) measurements are summarized in Table 1.

**Table 1:** Paired optic nerve sheath diameter (ONSD) and intracranial pressure (ICP) measurements.

Measurement	Left ONSD (mm)	Right ONSD (mm)	Mean ONSD (mm)	ICP (cmH₂O)	Intracranial Hypertension
1	–	4.90	4.90	27	No
2	6.18	5.50	5.84	27.5	Yes
3	6.29	6.41	6.35	45	Yes
4	6.89	5.97	6.43	31.5	Yes
5	6.80	6.46	6.63	41.5	Yes
6	6.20	6.50	6.35	50	Yes
7	6.80	6.46	6.63	43	Yes
8	5.40	4.50	4.95	34	Yes
9	5.95	6.45	6.20	22	No
10	6.44	5.95	6.20	50	Yes
11	5.00	4.50	4.75	16	No
12	7.45	6.95	7.20	55	Yes
13	6.32	5.70	6.01	25	No
14	7.62	7.84	7.73	37	Yes
15	5.42	5.60	5.51	16	No

Individual measurements of left and right eye optic nerve sheath diameter obtained by ultrasound and corresponding intracranial pressure values measured by lumbar puncture. Mean ONSD values were calculated when bilateral measurements were available.

No statistically significant difference was observed between right and left eye ONSD measurements (Wilcoxon test, *p* = 0.069). However, the near-significant *p*-value and the magnitude of discrepancy in some cases may reflect the operator-dependent nature of ONSD ultrasound. Small variations in probe positioning and measurement angle can lead to millimetric differences, particularly in pediatric bedside conditions. The absence of a consistent directional bias suggests that these differences are more likely technical rather than reflecting true anatomical asymmetry. Therefore, mean bilateral ONSD values were used for subsequent analyses.

Median ONSD was 6.20 mm (IQR 5.68–6.53), while median ICP was 34 cmH₂O (IQR 26–44).

**Figure 1:**
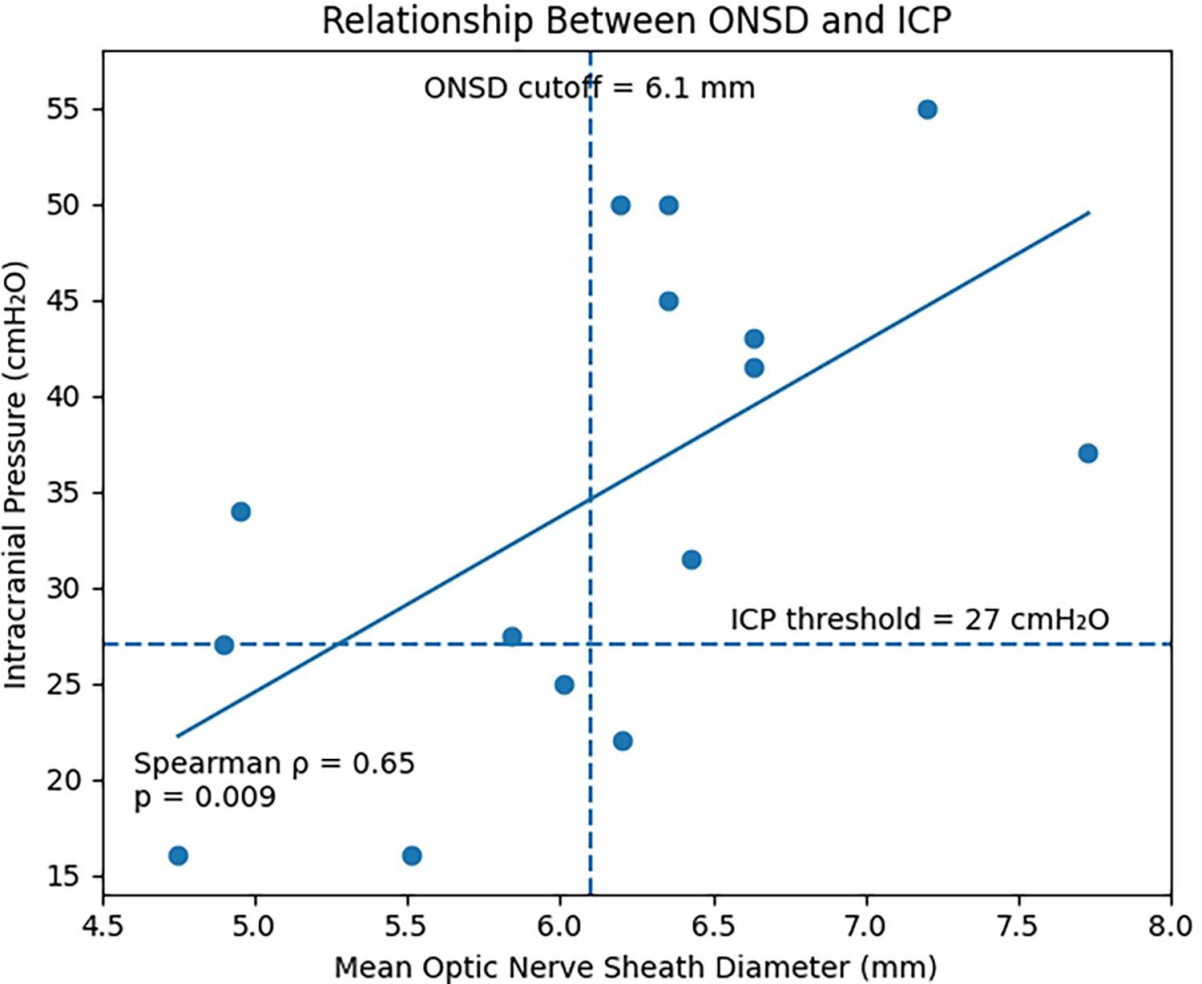
Relationship between optic nerve sheath diameter and intracranial pressure. Scatter plot showing the correlation between mean optic nerve sheath diameter (ONSD) measured by ultrasound and intracranial pressure (ICP) obtained by lumbar puncture. Each point represents one paired measurement. Dashed lines indicate the proposed ONSD cutoff value (6.1 mm) and the ICP threshold for intracranial hypertension (27 cmH₂O). A significant positive correlation was observed (Spearman *ρ* = 0.65, *p* = 0.009).

A significant positive correlation was observed between mean ONSD and ICP values (Spearman's rho = 0.65, *p* = 0.009). The relationship between ONSD and ICP is illustrated in Figure 1.

**Figure 2:**
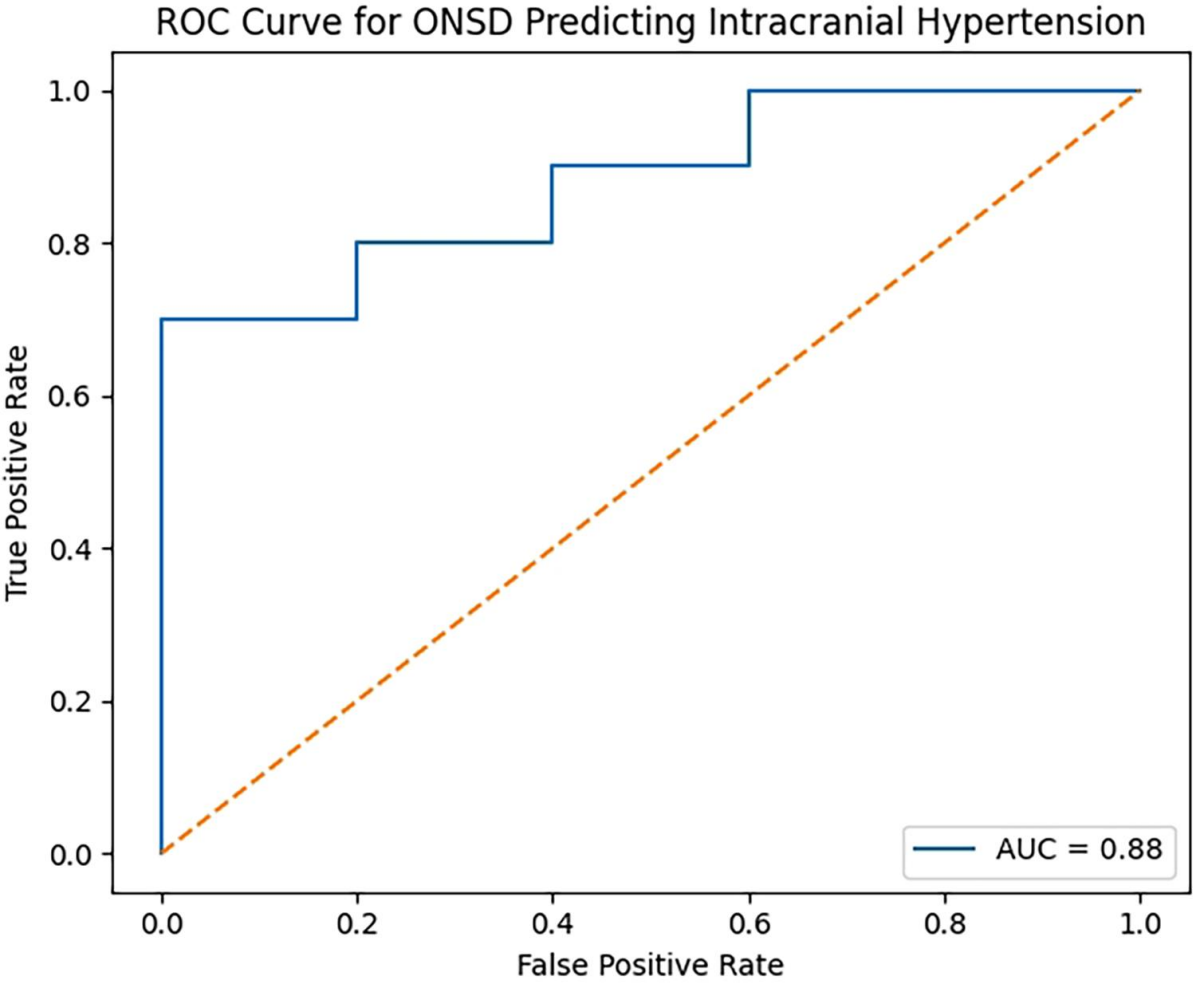
Receiver operating characteristic (ROC) curve for optic nerve sheath diameter in detecting intracranial hypertension. ROC curve illustrating the diagnostic performance of mean optic nerve sheath diameter (ONSD) measured by ultrasound for identifying intracranial hypertension (defined as ICP > 27 cmH₂O). The area under the curve (AUC) was 0.88, indicating good diagnostic accuracy.

Receiver operating characteristic (ROC) curve analysis identified an optimal ONSD cutoff value of 6.1 mm for detecting intracranial hypertension (defined as ICP > 27 cmH₂O), with a sensitivity of 80% and specificity of 80%. The area under the ROC curve (AUC) was 0.88 (95% CI 0.70–1.00). The ROC curve is shown in Figure 2.

An ONSD value greater than 6.1 mm was significantly associated with intracranial hypertension (*p* = 0.047), with an odds ratio of 16 (95% CI 1.09–234.25). Agreement between ONSD-based classification and direct ICP measurement was moderate (Cohen's *κ* = 0.57).

## Discussion

Our findings suggest that ultrasonographic ONSD measurement may be a useful non-invasive method for detecting intracranial hypertension in pediatric patients with closed fontanelles. The significant positive correlation observed between ONSD and directly measured ICP is consistent with previous studies demonstrating transmission of cerebrospinal fluid pressure from the intracranial compartment to the optic nerve sheath (5, 6).

Several adult studies have shown good diagnostic accuracy of ONSD ultrasound for detecting elevated ICP in critical care settings (7, 8). A large meta-analysis including both adult and pediatric populations reported high sensitivity and specificity for ONSD measurement in detecting intracranial hypertension, supporting its utility as a screening tool (9).

Pediatric data remain limited, and reported ONSD cutoff values vary widely across studies. Previous pediatric investigations have proposed thresholds ranging from 4.0 to 5.5 mm depending on patient age, ultrasound methodology, and reference standards used for intracranial pressure assessment (10–12). In older children with closed fontanelles, higher cutoff values have been described. For example, Padayachy et al. reported a threshold of 5.75 mm for identifying intracranial hypertension in children (12). The slightly higher cutoff value observed in our cohort (6.1 mm) may be explained by several factors, including differences in measurement technique, patient positioning, and the use of lumbar puncture opening pressure rather than continuous intraparenchymal monitoring as the reference standard. Additionally, the relatively older age of our cohort and the inclusion of repeated measurements in some patients may have contributed to the observed threshold. Despite these differences, our findings remain broadly consistent with previously published pediatric data.

The variability in proposed ONSD thresholds across studies likely reflects differences in patient age, ultrasound technique, anatomical landmarks used for measurement, and reference standards for ICP assessment. Importantly, our study used direct ICP measurement via lumbar puncture performed simultaneously with ultrasound examination, reducing temporal variability and strengthening the observed association. It should also be considered that optic nerve sheath enlargement may persist for hours to days after episodes of intracranial hypertension, suggesting that ONSD reflects recent or sustained ICP elevation rather than strictly instantaneous pressure levels.

ONSD ultrasound offers several advantages in pediatric intensive care, including bedside availability, repeatability, low cost, and avoidance of radiation exposure or invasive monitoring (13). These features make it particularly attractive for early screening of intracranial hypertension in clinically unstable patients or in settings where invasive monitoring is not immediately available.

However, the role of ONSD measurement in monitoring dynamic changes in ICP remains controversial. It should also be noted that our sample size is comparable to that of prior pediatric studies evaluating ONSD and intracranial pressure, including the study by Padayachy et al., which similarly included a relatively small cohort. This reflects the practical challenges of recruiting pediatric patients requiring simultaneous invasive ICP measurement.

Some studies suggest that ONSD normalization may lag behind acute reductions in ICP, limiting its utility for short-term monitoring (14). In contrast, other reports have demonstrated significant reductions in ONSD following therapeutic cerebrospinal fluid drainage, indicating potential responsiveness to acute ICP changes (15). Further prospective studies are needed to clarify this issue in pediatric populations.

### Limitations

This study has limitations. Its relatively small sample size and single-center design restrict statistical power and limit generalizability. However, this reflects the low incidence of pediatric intracranial hypertension and the strict inclusion criterion requiring simultaneous invasive ICP measurement, which substantially limits eligible cases. Similar sample sizes have been reported in previous pediatric studies in this field. In addition, intracranial pressure was measured using lumbar puncture rather than continuous intraparenchymal monitoring, which may introduce methodological variability. The results may therefore not be applicable to certain populations such as infants with open fontanelles or patients who have undergone decompressive craniectomy. Accordingly, these findings should be interpreted as preliminary and hypothesis-generating.

Interocular variability in ONSD measurements was observed in some cases, which may reflect the operator-dependent nature of the technique and subtle variations in probe positioning.

Because of the limited sample size and the exploratory nature of this pilot study, bilateral eye measurements were averaged rather than analyzed using mixed-effects modelling to account for within-patient correlation. Although mixed-effects models may provide a more sophisticated approach for handling correlated measurements, the small number of observations in this study limited the feasibility of such modelling.

## Conclusions

In this pilot study, ultrasonographic measurement of optic nerve sheath diameter appeared to be a feasible and reproducible non-invasive method for detecting intracranial hypertension in pediatric patients with closed fontanelles. An ONSD threshold of 6.1 mm may help identify intracranial hypertension, although larger multicenter studies are required to confirm these findings.

## Data Availability

The raw data supporting the conclusions of this article will be made available by the authors, without undue reservation.
